# Basal Ganglia Calcification With Treatment-Resistant Hypocalcaemia and Persistent Psychosis in an 18-Year-Old: A Case Report

**DOI:** 10.7759/cureus.95734

**Published:** 2025-10-30

**Authors:** Noor Abu Zar, Ashraf Elsaadany

**Affiliations:** 1 Acute Medicine, Queen Elizabeth Hospital, London, GBR

**Keywords:** adolescent seizure, basal ganglia calcification, brain calcification, fahr syndrome, hypocalcaemia, multi-hormonal resistance, organic psychosis, parathyroid hormone resistance, pseudohypoparathyroidism, treatment-resistant psychosis

## Abstract

Basal ganglia calcification (BGC) in young adults represents a pathological finding requiring systematic investigation, contrasting with incidental age-related calcification in elderly populations. We report an 18-year-old male patient who presented to the emergency department with a first episode of generalized tonic-clonic seizure with subsequent imaging revealing extensive bilateral BGC involving the basal ganglia, thalami, and cerebellum. His inpatient stay was complicated by treatment-resistant hypocalcaemia. A retrospective review revealed that hypocalcaemia had been documented eight months prior to this presentation. The patient had been suffering from new-onset psychotic symptoms beginning 12 months prior, which were poorly responsive to multiple antipsychotics and had initially been managed as primary functional psychosis. This presentation suggested that the neurological, psychiatric, and metabolic presentations are organically interlinked. Comprehensive screening for autoimmune encephalitis and infectious aetiologies was negative, with genetic testing for pseudohypoparathyroidism currently ongoing. Pseudohypoparathyroidism emerges as the leading hypothesis based on treatment-resistant hypocalcaemia, elevated parathyroid hormone (PTH) levels, and extensive calcification. The temporal sequence, psychosis (12 months) → documented hypocalcaemia (eight months) → seizure, suggests that long-standing calcium dysregulation and progressive BGC may have contributed to the psychiatric presentation, ultimately manifesting as seizure. This case demonstrates that investigating treatment-resistant psychiatric symptoms for organic causes and recognizing BGC in young patients as pathological red flags may facilitate earlier diagnosis before acute neurological complications.

## Introduction

Basal ganglia calcification (BGC) in young adults demands systematic investigation as it represents pathological rather than physiological findings. While age-related calcification may be incidental in elderly populations, occurring in up to 15-20% without clinical correlates [1], calcification in patients under 40 years should prompt comprehensive evaluation for underlying metabolic, genetic, or systemic causes [2].

The mean age of clinical onset for brain calcification syndromes is typically in the fourth or fifth decade, around 40-50 years (median: 43 years), making paediatric and adolescent presentations rare [3]. Secondary causes, often termed as Fahr's syndrome, include disorders of calcium-phosphate homeostasis, with hypoparathyroidism and pseudohypoparathyroidism being the most common causes of pathological BGC. These disorders may present with neurological complaints, including seizures, paraesthesia, depression, psychosis, and extrapyramidal manifestations [4]. Up to 74% of patients with idiopathic hypoparathyroidism develop brain calcifications that may be associated with psychiatric symptoms indistinguishable from primary psychiatric disorders [3].

We present a case illustrating the diagnostic complexity and systematic approach required when BGC occurs in a young adult with concurrent neuropsychiatric and metabolic manifestations, emphasizing recognition patterns that should prompt comprehensive organic investigation in the context of progressive metabolic abnormalities. 

## Case presentation

Clinical history 

An 18-year-old male patient presented to the emergency department via ambulance following his first witnessed generalized tonic-clonic seizure. The episode lasted approximately 10-15 minutes, characterized by generalized limb stiffening, eye rolling, and oral frothing, followed by prolonged post-ictal confusion and agitation. Evidence of tongue bite was present (dried blood on the inner lip), with no bowel or bladder incontinence reported.

The patient had no significant medical history before neuropsychiatric symptoms began 12 months prior. Four months after the onset of the symptoms, he presented to the emergency services with loss of appetite and nausea along with worsening mental health symptoms; he was subsequently referred to the Child and Adolescent Mental Health Services. His psychiatric manifestations included religious delusions, grandiose beliefs (claiming to be a prophet with "millions of babies"), and auditory hallucinations. He experienced persistent auditory hallucinations, reporting 5-6 distinct voices that were often perceived as instructive or commanding in nature (e.g., "go for a walk" or "wash yourself"). He found them difficult to ignore but generally not overtly distressing or harmful. These voices were often described as a constant commentary giving innocent commands, and observations by the psychiatry team noted both second- and third-person auditory hallucinations. His initial signs included social withdrawal, neglect of personal hygiene, declining academic performance, and stereotyped movements (such as bringing his hands up to his face as if praying), grimacing, and severely limited speech (one-word answers). Over the subsequent months, he experienced progressive deterioration with slurred speech, delayed responses, and increased dependence on assistance for basic self-care.

Six months after the onset of his psychotic symptoms, the patient was referred to the psychiatry team for further management. The treatment involved sequential trials of four antipsychotic medications. Initial treatment began with aripiprazole (started February 5; 5 mg titrated to 15 mg daily) for approximately two weeks. It was switched on February 19 to lurasidone (37 mg titrated to 55.5 mg daily), which was trialled for one month. Due to minimal response and side effects, treatment was switched on March 20 to olanzapine (15 mg daily). The patient was finally switched to clozapine (300 mg daily) on July 28, demonstrating persistent psychosis poorly responsive to these sequential agents over a six-month period. No dedicated neuroimaging was performed at the time of these psychiatric evaluations.

The patient had a family history of depression in both parents, including a psychotic episode in her 70s experienced by a paternal half-sister. There was no family history of epilepsy or seizure disorders. The patient denied any ongoing use of illicit substances, which was confirmed by a negative toxicology screening test.

Physical examination 

On assessment during the post-ictal phase, the patient presented with a Glasgow Coma Scale (GCS) score of 7 (E1/V1/M5), which later improved to 15/15 within the initial assessment period [5]. A bedside observation measurement demonstrated tachycardia (heart rate: 104-112 bpm), mild hypertension (blood pressure: 136/84 mmHg, normalizing to 123/70 mmHg), a respiratory rate of 12-14/min, an oxygen saturation of 96-98% on room air, and a temperature of 36.4-37.4°C. Evidence of recent seizure activity included dried blood on the inner aspect of the lower lip, consistent with a minor tongue bite, with no other injuries identified. The neurological examination showed bilaterally sluggish pupils at 4 mm (later equal and reactive at 3 mm), intact cranial nerves, reduced motor power in the upper limbs (4/5 bilaterally) with normal lower limb power (5/5), positive Babinski reflex on the right, and intact sensation throughout. The remainder of the physical examination, including the cardiovascular, respiratory, and abdominal systems, was unremarkable. 

General physical examination did not reveal features consistent with Albright hereditary osteodystrophy (AHO), such as short stature, round facies, or brachydactyly. The patient had no reported intellectual disability, history of eating disorders, or overt fatigue beyond what was associated with his post-ictal state and psychiatric decline.

Diagnostic investigations 

Table 1 demonstrates the temporal progression of laboratory values over eight months, revealing progressive metabolic deterioration with worsening hypocalcaemia and multi-hormonal abnormalities. The calcium levels declined from mildly low at his initial emergency presentation to a critically low value when he presented with his first tonic-clonic seizure event, with minimal improvement despite aggressive replacement therapy. Additional metabolic derangements included low free thyroxine and dyslipidaemia (low high-density lipoprotein (HDL) and elevated triglycerides), suggesting multi-hormonal resistance. Endocrine evaluation demonstrated elevated parathyroid hormone (PTH) with vitamin D insufficiency, elevated alkaline phosphatase, and normal phosphate, a pattern consistent with secondary hyperparathyroidism. Serial magnesium levels checked throughout this period remained within the normal range (0.7-1 mmol/L), helping to exclude functional PTH resistance due to hypomagnesaemia. Furthermore, an electrocardiogram (ECG) performed on admission revealed no acute changes, specifically confirming a normal QTc interval. Complete blood count and renal and liver function were within normal limits. 

**Table 1:** Serial laboratory investigations demonstrating progressive metabolic dysfunction This table presents the key laboratory parameters at four time points spanning eight months prior to seizure presentation through hospital discharge. Values outside the reference range demonstrate progressive hypocalcaemia, multi-hormonal abnormalities, and treatment resistance despite aggressive supplementation. - indicates the test was not performed at that time point. TSH: thyroid-stimulating hormone; HDL: high-density lipoprotein

Parameter	Unit	Reference range	8 months pre-admission	1 month pre-admission	Admission	On discharge
Adjusted calcium	mmol/L	2.2-2.6	1.9	-	1.51	2.02
Phosphate	mmol/L	0.8-1.5	1.66	-	1.49	1.72
Parathyroid hormone	pmol/L	1.6-6.9	-	-	32.3	-
25-OH vitamin D	nmol/L	50-250	-	-	37	-
Free T4	pmol/L	12-22	-	9.5	8	-
TSH	mU/L	0.27-4.2	-	3.67	2.77	-
HDL cholesterol	mmol/L	>1	-	0.7	-	-
Triglycerides	mmol/L	<1.7	-	4.83	-	-

A comprehensive autoimmune encephalitis screening came back negative and encompassed tests for NMDA receptor, voltage-gated calcium and potassium channels, and CASPR2, LGI-1, AMPA-1, AMPA-2, and GABA-B receptor antibodies. Testing for glycine receptor antibodies and paraneoplastic antibodies (Hu, Ri, Yo) and genetic testing for primary familial brain calcification and pseudohypoparathyroidism all remained pending at discharge. Infectious disease screening for hepatitis B and C, human immunodeficiency virus (HIV), and syphilis was negative.

Cerebrospinal fluid (CSF) analysis yielded clear colourless fluid with a white cell count of <3/mm^3^, a red cell count of 345/mm^3^ (likely traumatic tap), and a markedly elevated protein (678 mg/L; normal: 150-400 mg/L). CSF glucose was 2.9 mmol/L with a paired plasma glucose of 5.1 mmol/L (ratio 0.57, borderline low). Gram stain showed no organisms, with negative bacterial culture after 48 hours. 

Neuroimaging 

**Figure 1:**
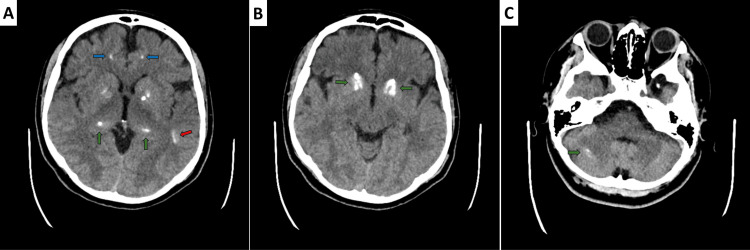
Non-contrast CT of the head, axial view, showing extensive brain calcifications (A) The blue arrow indicates the calcification in the subcortical white matter of the frontal lobe; the green arrows highlight the bilateral calcification in the thalami; and the red arrow points to the calcification in the temporal lobe subcortical white matter. (B) The dense, bilateral calcification within the basal ganglia. (C) The calcification within the cerebellum. CT: computed tomography

Computed tomography (CT) of the head revealed extensive bilateral calcifications involving the basal ganglia (globus pallidus and putamen), thalami, subcortical white matter in the frontal and temporal lobes, and cerebellar hemispheres with the dentate nuclei (Figure 1). A 3 cm left temporal arachnoid cyst was incidentally noted. No acute pathology, haemorrhage, mass effect, midline shift, or skull fracture was identified. 

**Figure 2:**
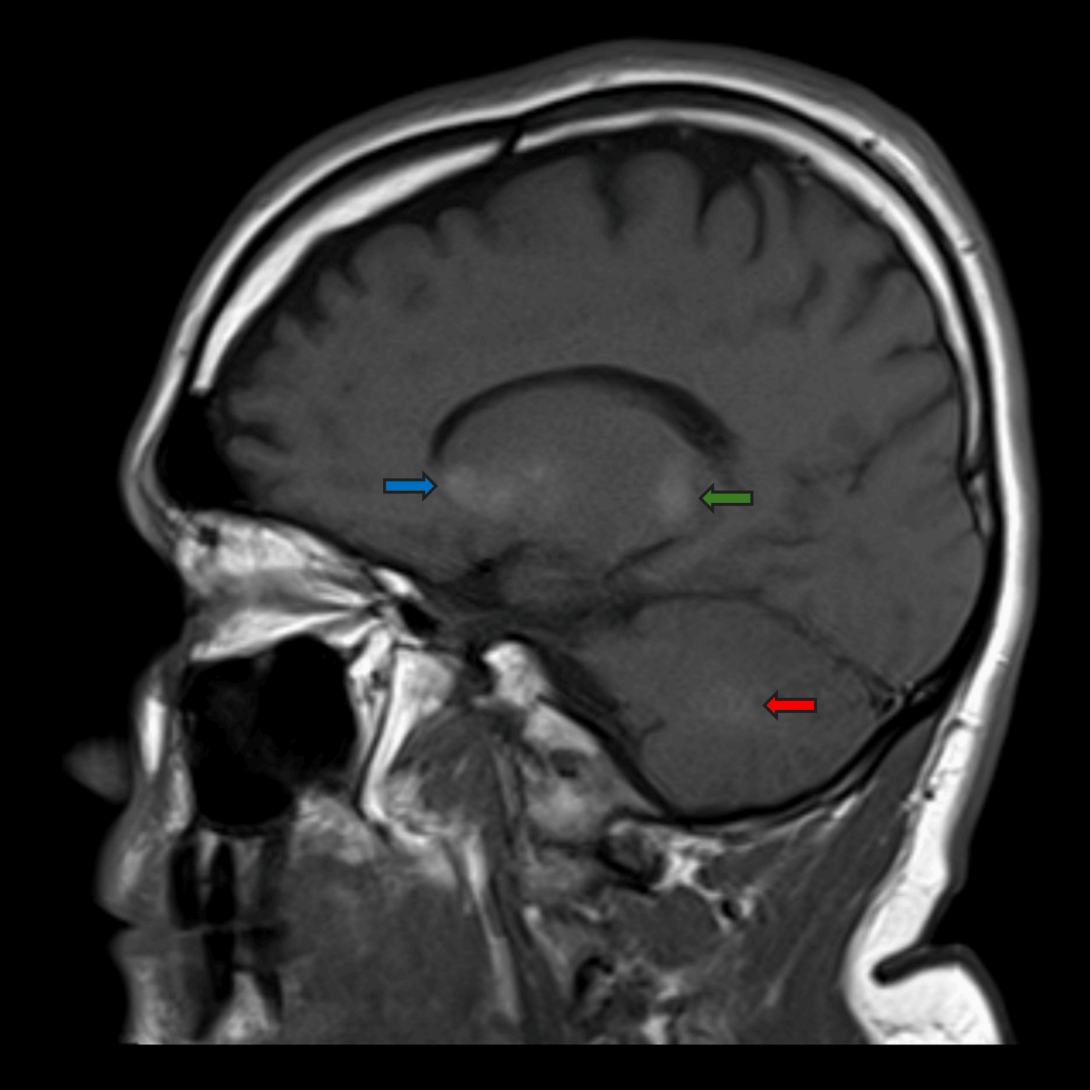
Non-contrast MRI of the head, sagittal section ​​​The sagittal T1-weighted image confirms the widespread mineralization seen on CT, visible as areas of hyperintensity (bright signal). Blue arrow: calcification within the basal ganglia; green arrow: calcification within the thalamus; red arrow: calcification within the cerebellum CT: computed tomography; MRI: magnetic resonance imaging

Magnetic resonance imaging (MRI) of the head with gadolinium contrast confirmed symmetric deep hemispheric and subcortical mineralization appearing as T1 hyperintensity with faint susceptibility artifact (Figure 2). Brain volume was normal for age with appropriately sized ventricles and extra-axial CSF spaces. No pathological mass lesion, inflammatory changes, ischemic injury, or contrast enhancement was demonstrated.

Hospital course and initial management 

The patient was admitted and monitored by a multidisciplinary team (acute medicine, neurology, psychiatry liaison, and endocrinology) for the rest of his inpatient stay. No further seizure activities occurred during admission. The neurology team recommended withholding the antipsychotic medication until organic causes were ruled out. The psychiatry team observed stable psychotic symptoms despite the discontinuation of antipsychotic treatment, suggesting an organic rather than a primary psychiatric aetiology.

Immediate treatment included intravenous calcium gluconate followed by oral calcium and vitamin D. Despite aggressive replacement therapy, hypocalcaemia persisted throughout admission, improving minimally from 1.51 mmol/L to 2.02 mmol/L at discharge (still subnormal). The endocrinology team confirmed secondary hyperparathyroidism likely due to vitamin D deficiency but noted that persistent treatment-resistant hypocalcaemia raised suspicion for pseudohypoparathyroidism, with genetic confirmation pending.

The patient remained clinically stable with stable psychotic symptoms, primarily auditory hallucinations (reporting 5-6 voices with innocent commands or constant commentary) and a continued tendency toward social withdrawal and short, fast answers. No paranoid thoughts or delusions were elicited on discharge assessment, and no recurrent seizures occurred. He was discharged on low-dose risperidone 1 mg daily for symptomatic management with comprehensive outpatient follow-up, including neurology, endocrinology, and psychiatry services. Post-discharge, the phosphate levels normalized, but calcium remained persistently low despite uptitration to maximum-dose calcium supplementation, ultimately requiring the addition of alfacalcidol 0.5 mcg daily. Psychotic symptoms subsequently worsened acutely in the days immediately following discharge, necessitating community home treatment team involvement and medication re-evaluation. The worsening was characterized by severe agitation (shouting, hitting walls, breaking a door) and an increase in auditory hallucinations, which became sexually explicit and intrusive for the patient. The complex chronological evolution of the patient's symptoms, management, and ultimate diagnostic shift is visually summarized in Table 2.

**Table 2:** Timeline illustrating the temporal evolution of symptoms and diagnostic findings CT: computed tomography; BGC: basal ganglia calcification; PTH: parathyroid hormone; PHP: pseudohypoparathyroidism

Time point	Event/clinical finding	Diagnostic classification at the time
12 months pre-admission	Onset of neuropsychiatric symptoms (e.g., auditory hallucinations, grandiose ideas, social withdrawal)	Managed as primary functional psychosis
8 months pre-admission	First documented hypocalcaemia (1.9 mmol/L)	Metabolic finding likely attributed to poor intake
6 months pre-admission	Initiation of sequential antipsychotic trials	Persistent psychosis, poorly responsive to medication
Admission (0 months)	Generalized tonic-clonic seizure; critically low calcium (1.51 mmol/L); urgent CT scan performed	Acute neurological/metabolic crisis
Post-admission workup	Discovery of extensive BGC on CT; elevated PTH; low free T4	Hypocalcaemia and BGC confirmed; diagnostic shift to pathological organic aetiology
Discharge/follow-up	Stable psychosis (off medication); persistent hypocalcaemia requiring alfacalcidol; genetic testing pending	Leading hypothesis: PHP

## Discussion

BGC should be considered pathological in patients under 40 years, particularly when extensive or associated with clinical symptoms. This age-dependent significance reflects fundamentally different aetiologies compared to incidental age-related calcification in the elderly and requires systematic investigation. Our 18-year-old patient represents one of the youngest reported adult presentations of bilateral BGC with concurrent seizure and persistent psychosis, notable for documented progressive metabolic deterioration over eight months preceding acute neurological presentation. 

Temporal progression and diagnostic evolution 

The temporal sequence in this case, primary functional psychosis (12 months) → documented hypocalcaemia 1.9 mmol/L (eight months) → additional metabolic abnormalities including low T4 → seizure with critical hypocalcaemia 1.51 mmol/L, illustrates insidious metabolic progression with potential organic contribution to psychiatric symptoms. Our patient bridges typical age-related patterns with established psychosis, followed by generalized seizure. A previously documented 17-year-old presented with focal seizures and similar metabolic abnormalities [6], while adult presentations typically manifest psychiatric symptoms with movement disorders developing later [7]. Brain calcification in young patients demonstrates various clinical symptoms, including movement disorders, cognitive impairment, and psychiatric disturbances that may occur in isolation or combination [8]. 

Differential diagnosis and multi-hormonal resistance 

The combination of treatment-resistant hypocalcaemia, extensive bilateral calcification, seizure, and persistent psychosis in an adolescent patient requires the systematic evaluation of multiple potential aetiologies. 

Pseudohypoparathyroidism

Pseudohypoparathyroidism emerges as the leading hypothesis. Treatment-resistant hypocalcaemia requiring escalation to alfacalcidol, along with elevated PTH and an extensive calcification pattern, strongly supports end-organ resistance rather than simple deficiency states. Additional metabolic abnormalities documented pre-seizure, including low free T4 (9.5 pmol/L) and dyslipidaemia (low HDL 0.7 mmol/L, elevated triglycerides 4.83 mmol/L), suggest multi-hormonal resistance characteristic of pseudohypoparathyroidism type 1a, where GNAS mutations cause resistance to multiple G protein-coupled hormones, including PTH and thyroid-stimulating hormone (TSH). This multi-hormonal resistance pattern, combined with progressive calcium deterioration over eight months, supports end-organ resistance rather than isolated nutritional deficiency. The co-occurrence of hypocalcaemia, hypothyroidism, and dyslipidaemia in a young patient should specifically raise suspicion for pseudohypoparathyroidism type 1a, as this clustering reflects multi-hormonal G protein-coupled receptor resistance rather than multiple independent deficiencies [9]. BGC in these disorders results from the chronic deposition of calcium-phosphorus complexes in the cerebral tissue, with both local and systemic metabolic mechanisms contributing to calcification development [10]. The neuropsychiatric symptoms and seizures can be explained through calcium-mediated neuronal dysfunction affecting ion channels and neurotransmitter release [11], combined with the structural effects of BGC. Extrapyramidal manifestations, specifically rigidity and tremors, are well-documented features in case reports of hypoparathyroidism with BGC [12]. *Primary Familial Brain Calcification*

Primary familial brain calcification, historically known as Fahr's disease, cannot be excluded despite absent family history, which occurs in up to 40% of cases due to variable penetrance and de novo mutations [3]. Seven known causative genes can present with neuropsychiatric symptoms, including psychosis, cognitive decline, and seizures, in young adults. Psychiatric and behavioural problems occur in 20-30% of people with primary familial brain calcification, including difficulty concentrating, memory loss, personality changes, psychosis, and cognitive decline, with the extent of calcification potentially influencing symptom severity [8]. The extensive calcification pattern involving the basal ganglia, thalami, and cerebellar structures is consistent with primary familial brain calcification's radiological phenotypes. However, the biochemical profile of progressive hypocalcaemia with elevated PTH and multi-hormonal abnormalities makes secondary causes, or Fahr's syndrome, more likely. *Secondary Metabolic and Autoimmune Processes*

Secondary metabolic and autoimmune processes were comprehensively screened. Secondary metabolic causes were identified, including vitamin D deficiency and secondary hyperparathyroidism, though incomplete response to supplementation and persistent severe hypocalcaemia suggest additional pathology beyond simple nutritional deficiency. Autoimmune encephalitis antibodies returned negative, though elevated CSF protein (678 mg/L) warrants the consideration of chronic low-grade neuroinflammation secondary to calcification or blood-brain barrier dysfunction from chronic hypocalcaemia.

Clinical recognition patterns and diagnostic red flags 

This case demonstrates five critical clinical, metabolic, and radiological red flags that necessitate a comprehensive organic workup in young adults presenting with neuropsychiatric symptoms. The clustering of these features, particularly the treatment-resistant psychosis and progressive hypocalcaemia alongside extensive BGC, strongly suggests an underlying systemic pathology (e.g., pseudohypoparathyroidism) rather than primary psychiatric illness (Table 2). 

**Table 3:** Diagnostic red flags guiding the organic investigation of BGC BGC: basal ganglia calcification

Red flag category	Clinical features	Supporting evidence from this case	Clinical action/rationale
Multiple antipsychotic failures	Minimal response to ≥2 agents [13], particularly with rapid titration and poor tolerance	4 agents in 6 months (aripiprazole, lurasidone, olanzapine, clozapine) with persistent symptoms	Requires comprehensive organic workup; high treatment resistance suggests underlying structural or systemic pathology
Atypical psychosis presentation	Early onset (≤18 years), rapid functional decline, motor/catatonic features, new seizures [14]	Onset age 17, progressive decline with slurred speech, motor slowing, catatonia-like episodes	Demand for urgent investigation; divergence from primary psychotic features suggests secondary/organic cause
Metabolic abnormalities	Hypocalcaemia (<2 mmol/L) or clustering of endocrine abnormalities (thyroid, lipid) [14]	Ca 1.9 mmol/L (8 months pre-seizure), low T4 9.5 pmol/L, dyslipidaemia	Pathophysiological clue; indicates multi-systemic endocrine derangement (e.g., pseudohypoparathyroidism)
Temporal progression	Sequential evolution: psychosis → metabolic changes → neurological event	12 months psychosis → 8 months documented hypocalcaemia → seizure	Concerning symptom sequence with metabolic abnormalities that requires early recognition and prompt investigation
BGC in young adults	Extensive bilateral calcification in patients <40 years [1]	18-year-old with extensive bilateral involvement of the basal ganglia, thalami, and cerebellum	Definitive pathological finding; BGC at this age is not incidental and mandates investigation for calcium/endocrine disorders

## Conclusions

This case demonstrates the critical importance of recognizing BGC as pathological in young adults under 40 years. The constellation of progressive, treatment-resistant hypocalcaemia, extensive calcification, multi-hormonal abnormalities, seizure, and persistent psychosis in an 18-year-old necessitates systematic investigation for metabolic and genetic causes. Pseudohypoparathyroidism emerges as the leading hypothesis based on a multi-hormonal resistance pattern and treatment-resistant hypocalcaemia requiring alfacalcidol, with genetic confirmation pending. Despite a comprehensive workup ruling out common autoimmune and infectious aetiologies, the definitive diagnosis hinges on the pending genetic analysis, underscoring the limitations of standard acute investigation in complex neuroendocrine disorders. This case highlights several critical clinical principles, starting with the need for investigating treatment-resistant psychiatric symptoms for organic causes, particularly when accompanied by metabolic abnormalities. It stresses the importance of recognizing documented metabolic derangements in neuropsychiatric presentations, as these findings warrant comprehensive investigation. Furthermore, clinicians should utilize temporal relationship analysis to distinguish evolving organic pathology from a primary psychiatric illness and must maintain diagnostic flexibility before establishing a final diagnosis of treatment-resistant psychiatric illness. Ultimately, this case emphasizes that early recognition and systematic investigation of metabolic abnormalities alongside neuropsychiatric symptoms may facilitate the earlier diagnosis of underlying organic disease before progression to acute neurological complications. 
